# A Milk Foodomics Investigation into the Effect of *Pseudomonas fluorescens* Growth under Cold Chain Conditions

**DOI:** 10.3390/foods10061173

**Published:** 2021-05-24

**Authors:** Paolo Bellassi, Gabriele Rocchetti, Lorenzo Morelli, Biancamaria Senizza, Luigi Lucini, Fabrizio Cappa

**Affiliations:** Department for Sustainable Food Process (DiSTAS), Università Cattolica del Sacro Cuore, via Emilia Parmense 84, 29122 Piacenza, Italy; gabriele.rocchetti@unicatt.it (G.R.); lorenzo.morelli@unicatt.it (L.M.); biancamaria.senizza@unicatt.it (B.S.); fabrizio.cappa@unicatt.it (F.C.)

**Keywords:** milk contamination, spoilage, untargeted profiling, foodomics, molecular marker, milk quality

## Abstract

*Pseudomonas fluorescens* is a psychrotrophic species associated with milk spoilage because of its lipolytic and proteolytic activities. Consequently, monitoring *P. fluorescens* or its antecedent activity in milk is critical to preventing quality defects of the product and minimizing food waste. Therefore, in this study, untargeted metabolomics and peptidomics were used to identify the changes in milk related to *P. fluorescens* activity by simulating the low-temperature conditions usually found in milk during the cold chain. Both unsupervised and supervised multivariate statistical approaches showed a clear effect caused by the *P. fluorescens* inoculation on milk samples. Our results showed that the levels of phosphatidylglycerophosphates and glycerophospholipids were directly related to the level of contamination. In addition, our metabolomic approach allowed us to detect lipid and protein degradation products that were directly correlated with the degradative metabolism of *P. fluorescens*. Peptidomics corroborated the proteolytic propensity of *P. fluorescens*-contaminated milk, but with lower sensitivity. The results obtained from this study provide insights into the alterations related to *P. fluorescens* 39 contamination, both pre and post heat treatment. This approach could represent a potential tool to retrospectively understand the actual quality of milk under cold chain storage conditions, either before or after heat treatments.

## 1. Introduction

Raw milk microbiota are susceptible to changing during milk transformation into dairy products (e.g., fresh cheese, drinking-thermal-treatment milk), which can impair both the quality and safety of the final product [1]. The cold chain becomes of great importance for keeping the product as stable as possible. Raw milk is usually kept in a refrigeration temperature range (0–10 °C; REG. CE 853/2004) until it is processed, but refrigeration is not enough to prevent psychrophilic and psychrotrophic microorganisms from growing at low temperatures.

The thermal treatments that typically characterize milk processing (e.g., pasteurization or sterilisation) eliminate the psychrotrophic community but do not inactivate several degradative enzymes that can affect and damage milk during its shelf life [2]. For example, the gelification of ultra-high-temperature-processed (UHT) milk is a common problem related to heat-stable proteolytic activity on milk proteins following the heat treatment. Also problematic are the lipolytic reactions resulting from heat-resistant lipases that persist in the milk, thus generating undesirable off-flavours [3].

Among psychrotrophic microorganisms, members of the genus *Pseudomonas* have extensive genetic diversity and metabolic versatility, which allow them to survive in different environments, such as soil, water and air [4]. In the *Pseudomonas* genus, the species *P. fluorescens* has many strains adapted to the milk environment that possess psychrotrophic characteristics that allow it to grow at refrigeration temperatures [5]. Additionally, it produces a number of exoenzymes that can contribute to the deterioration of raw milk [6,7]. Therefore, all these characteristics allow them to survive on the tools and equipment used throughout the milk production chain (e.g., pipelines, bulk transport tanks, milking machines and the animal production environment). Overall, *P. fluorescens*, *P. aeruginosa* and *P. putida* are the main *Pseudomonas* species present in the dairy chain [8]. These microorganisms produce proteolytic and lipolytic enzymes resistant to high temperatures, thus causing milk deterioration, premature coagulation and off-flavours, even in processed dairy products [9,10].

In this regard, numerous culture-dependent and culture-independent methods (e.g., those based on specific DNA detection) have been developed to detect the presence of *Pseudomonas* in milk [11,12,13]. Culture-dependent methods require live cells and are only applicable to milk prior to heat treatment. In contrast, culture-independent methods based on DNA detection can also detect the status of past contamination, even post heat treatment.

Foodomics represents a powerful tool for determining the food constituents and nutrients at the molecular level. In the last few years, foodomics studies have been realized by using different analytical approaches and strategies, such as those based on different omics disciplines such as proteomics, metabolomics, lipidomics, nutrigenomics, metagenomics and transcriptomics [14]. Accordingly, untargeted metabolomics and peptidomics are potentially able to provide useful insights into the transformations associated with biological activity at a given time and under certain biological or environmental conditions [15]. This approach can be useful for detecting activity related to either the current or previous presence of *Pseudomonas*, thus predicting possible quality-related degradation phenomena occurring in milk [16]. Indeed, metabolomic approaches have shown promising results in various areas, including the search for *P. aeruginosa* in the medical field [17]. Still, *Pseudomonas*-related activity and its effect at the molecular level in milk are not yet fully understood.

Therefore, in this preliminary study, although focusing attention on a low number of samples, we aim to simulate a real-case scenario to better understand the chemical and biochemical alterations induced by *Pseudomonas* in milk, considering potential contamination occurring before thermal treatments. In this regard, our purpose is to unravel preliminary milk quality markers that correlate with (either present or past) contamination by *Pseudomonas*, focusing on the concept of “quality” perceived from a wide perspective, and not only from a microbiological point of view. To this aim, both polar and lipophilic metabolites were considered, and markers of microbial growth were investigated either before or after thermal treatment in order to achieve more realistic conditions. Such information could represent a valuable tool to be used by the dairy industry in order to investigate the metabolic signature induced by a *Pseudomonas* contamination in milk, even in the context of retrospective inspections of previous contamination, thus providing somewhat complementary information to microbiological assays. Finally, our aim is to highlight the potential of a multiomics approach in dairy science and to further extend the present findings to a larger dataset.

## 2. Materials and Methods

### 2.1. Strain Growth Conditions

The wild strain used in the experiment was *P. fluorescens* 39, originally isolated from raw milk and provided from the collection of the DiSTAS Department (Università Cattolica del Sacro Cuore, Piacenza, Italy). The inoculum was prepared by reactivating the frozen strain using Nutrient Broth (NB; Thermo Fisher Scientific™, Oxoid™, Waltham, MA, USA) culture medium at 30 °C for 48 h. During the experimental test, *P. fluorescens* 39 was grown at 6 °C in ultra-high temperature (UHT) milk and 6 °C in semi-skimmed, microfiltered milk. A selective medium specifically for the *Pseudomonas* count was used to measure growth (*Pseudomonas*-Agar base; PAB; Thermo Fisher Scientific™, Oxoid™, USA) supplemented with a specific mix containing 5 mg cetrimide, 5 mg fusidic acid and 25 mg cephalosporin according to ISO/TS 11059:2009.

### 2.2. Experimental Plan

The inoculum was prepared as described by Stoeckel et al. [18]. The strain was reactivated from the stock frozen at −20 °C in NB at 30 °C for 24 h and reinoculated twice consecutively to obtain viable biomass. The cell suspension was then centrifuged (5000 rpm, 10 min). The pellet was washed with saline buffer (9 g/L NaCl) and resuspended in partially skimmed UHT milk incubated at 6 °C for 6 days to allow the bacteria to adapt to the milk medium and cold conditions. The final concentration was ∼10^8^ CFU/mL [19]. Subsequently, it was inoculated in triplicate (n = 3) to build three separate experiments with a final concentration of about ~10^4^ CFU/mL in commercially available microfiltered milk. This condition was preliminarily tested for the native presence of endogenous contamination by *Pseudomonas* or other microorganisms using a selective PAB medium and non-selective MPCA medium. Microfiltered milk was chosen as a model substrate primarily because it better simulates the physical-chemical state of the milk in the collection tanks before being processed. In addition, microfiltration and pasteurization allowed us to have practically quasi-sterile milk and reduced the possibility of indigenous milk microorganisms that could interfere with the experimental test [19]. The samples were then incubated for 24 h and 144 h, respectively, at 6 °C. The two periods of incubation were chosen to reach two different levels of contamination, one close to the legal limit of 10^5^ CFU/mL, and one corresponding to excessive microbial growth, namely 10^8^ CFU/mL. Subsequently, samples at both time points were divided into four aliquots; two aliquots were directly extracted for metabolomics analysis (see Section 2.3). The other two aliquots were thermally treated at 97 °C for 4 min [19] before metabolites extraction. At the same time, the control milk samples underwent the same experimental processes previously described, without inoculation. All samples were stored in frozen conditions until analysis.

### 2.3. Extraction of Metabolites from Milk

The extraction process was carried out as previously reported by [20], with solvent modifications. The frozen samples were thawed and then subjected to an extraction process based on two different extraction solvents: methanol and dichloromethane. To this aim, milk samples were skimmed by centrifugation at 4500× *g* for 10 min at 4 °C, then a 5 mL aliquot was put in contact with 10 mL of a methanol solution acidified with 3% formic acid. In parallel, another 5 mL aliquot of milk was mixed with 10 mL of dichloromethane acidified with 3% formic acid. Both extraction mixtures were centrifuged at 5000× *g* for 15 min at 4 °C (Thermo Scientific SL 40FR). Subsequently, the recovered supernatant was dried under a nitrogen stream, then resuspended in 1 mL of acetonitrile (LC-MS Chromasolv, ≥99.9% purity, Sigma-Aldrich) and filtered using a 0.22 µm cellulose membrane into amber vials for metabolomic analysis.

### 2.4. Ultra-High-Pressure Liquid Chromatography Coupled with Quadruple-Time-of-Flight Untargeted Metabolomics

The metabolomic profile of the milk samples inoculated with *P. fluorescens* 39 and the corresponding non-inoculated samples was performed using ultra-high-pressure liquid chromatography (UHPLC; Agilent 1200 series) with quadruple-time-of-flight (QTOF; Agilent 6550 iFunnel) detection. The instrumental conditions for the metabolomic analysis of the milk samples were optimised in a previous work [21]. Chromatography was designed, adopting a water-acetonitrile (both LCMS grade, from Sigma-Aldrich, Milan, Italy) binary gradient elution (6–94% acetonitrile in 32 min; 0.1% HCOOH as phase modifier) and using an Agilent Zorbax Eclipse Plus C18 column (50 mm × 2.1 mm, 1.8 μm). The injection volume was 6 μL for each sample. The sequence injection was randomised, and blank samples were injected to eliminate background features. An Agilent JetStream ESI ionisation source was used, adopting the previously optimised parameters [22]. The milk extracts were analysed in positive polarity following a Full-Scan mode in the range 100–1200 *m/z* (0.8 spectra/s, extended dynamic range mode, nominal mass resolution of 30,000 FWHM). The raw mass features were then processed according to the targeted “find-by-formula” algorithm with Agilent Profinder B.06 software (Agilent Technologies, Santa Clara, CA, USA). The combination of a monoisotopic accurate mass with an isotopes profile (both accurate isotopic spacing and ratio), and 5-ppm tolerance for mass accuracy, allowed the features to be annotated following mass and retention time alignment. To this end, the comprehensive *P. aeruginosa* metabolome database (PAMDB; [23]) plus amino acids, peptides and analogues, as well as lipids and lipid-like molecules, was used as a reference for annotation. Post-acquisition filtering was performed in Profinder B.06, retaining the compounds that passed the desired frequency of detection (within 100% of replications in at least one treatment). According to our annotation process, a Level 2 accuracy in identification (i.e., the putatively annotated compounds) was achieved, as reported by the COSMOS Metabolomics Standards Initiative [24].

### 2.5. Milk Peptidomics Profiling

Peptide extraction was initially performed by filtering milk samples through a 10 kDa Amicon filter (MilliporeSigma, Burlington, MA, USA) according to the manufacturer’s instructions to eliminate the predominant protein component of milk and isolate the peptides from the proteins. The filtrate was centrifuged at 7500× *g* for 30 min at 4 °C to eliminate the precipitated salts.

The protein content in the filtrate was quantified by a Qubit™ Protein Assay Kit (Life Technologies, Carlsbad, CA, USA), then 50 µg of peptides was transferred to a new tube, and 50 mM ammonium bicarbonate was added, up to a final volume of 100 µL. The reduction phase was then performed by adding 3 µL of Dithiothreitol (DTT), incubating at 56 °C for 40 min. Alkylation was then carried out by adding 3 µL of iodoacetamide (IAA) and incubating 40 min at room temperature. The extracts were quenched by adding 3 µL of DTT, and the digestion phase was performed by adding 2 µL 0.5 µg/µL trypsin at 37 °C overnight. The resultant peptides were analysed using nanoLC and quadruple-time-of-flight (Q-TOF) mass spectrometry, as previously reported [25]. An Agilent 1260 Chip Cube nanoLC coupled to an Agilent 6550 IFunnel Q-TOF mass spectrometer (Agilent Technologies, Santa Clara, CA, USA) was used. A 150 mm separation column (Zorbax 300SB-C18, 5 µm pore size) was used, and peptides were separated during a 150 min acetonitrile gradient (from 3% to 70% *v/v*) in 0.1% (*v/v*) formic acid at 0.3 µL min^−1^. The acquisition was carried out in positive polarity and a data-dependent mode (20 precursors per cycle), in the range 300–1700 *m/z*^+^. Peptide inference was produced from MS/MS spectra in Agilent SpectrumMill B.04, using carbamidomethylation of cysteine as a fixed modification, accepting one missed cleavage and the proteome of *Bos taurus* (Uniprot, accessed June 2019). Label-free quantification was conducted, and the false discovery rate was set to 1%.

### 2.6. Statistical Analysis

The raw data were then processed in a Mass Profiler Professional B.12.06 (Agilent Technologies, Santa Clara, CA, USA). Overall, metabolites were normalised at the 75th percentile, Log2-transformed and then baselined to the median value in all samples.

For metabolomic patterns, an unsupervised hierarchical cluster analysis (HCA) was initially carried out using MetaboAnalyst [26], setting the similarity measurement as “Euclidean” and “Wards” as the linking rule considering all compounds in the dataset. Thereafter, the raw dataset was exported to SIMCA 14.1 (Umetrics, Malmo, Sweden), Pareto-scaled and processed with orthogonal projections to latent structures discriminating analysis (OPLS-DA)-supervised modelling. Model parameters R^2^Y(cum) and Q^2^(cum) were calculated, with a threshold for Q^2^ prediction ability of >0.5, according to the recommendations of both the software and the literature [27]. In addition, the OPLS-DA models were cross-validated using CV-ANOVA (*p* < 0.01), while permutation tests (N = 100) were performed to exclude overfitting. Then, to elucidate the accumulation trends of discriminating metabolites, fold-change analysis was also performed (cut-off value ≥2) using Mass Profiler Professional (Agilent, Santa Clara, CA, USA). Finally, a Venn diagram was produced from up-accumulated metabolites considering the different incubation periods (i.e., 24 vs. 144 h). Only those compounds that were found to be common between the two incubation periods were retained because they were considered characteristic of *P. fluorescens* 39, irrespective of the time course. Finally, a volcano plot analysis was performed in MetaboAnalyst to identify differential metabolites at each contamination level, using a default cut-off FC value >2 and an FDR-adjusted *p*-value < 0.05. The resulting list of marker compounds was reinforced by variable of importance in projection (VIP) analysis on the OPLS-DA model, using the inoculated samples incubated for 24 and 144 h at 6 °C. Finally, differential metabolites were subjected to chemical similarity enrichment analysis (ChemRICH) as previously described [28]. To this aim, the online tool (ChemRICH, accessed March 2020) was used, and metabolites were grouped by chemical class according to the information reported in the PAMDB [23] by non-overlapping Tanimoto substructure chemical similarity coefficients.

## 3. Results and Discussion

### 3.1. Growth Capacity under Psychrotrophic Conditions

The number of colony-forming unit per millilitre (CFU/mL) of *P. fluorescens* 39 and possible contaminants was monitored at the time of inoculation, after 24 h under psychrotrophic conditions, to maintain CFU/mL around the legal limit (10^5^ CFU/mL; REG. CE 853/2004). In addition, a 144 h sample was tested to simulate the worst-case scenario of unprocessed milk being stored beyond normal industrial practices before sterilisation. The non-inoculated sample was negative on both *Pseudomonas*-selective medium and MPCA medium for total bacterial count (TBC) enumeration. By contrast, the inoculated sample had a *Pseudomonas* logarithmic CFU/mL of ~4 log_10_(CFU/mL), which then increased to ~5 log_10_(CFU/mL) after 24 h, while the 144 h sample had contamination of ~9 log_10_(CFU/mL). In addition, TBC on the treated samples reflects the deliberate inoculation of *P. fluorescens* 39 (Table 1). Maintaining these *Pseudomonas* concentrations was essential to properly simulate a realistic scenario concerning milk contamination during cold chain storage. Heat-treated samples (97 °C for 4 min) were, as expected, negative on the count both with selective *Pseudomonas* medium and with non-selective MPCA medium.

### 3.2. Untargeted Metabolomic Discrimination of the Different Milk Samples

Overall, 4071 compounds were annotated for each group of comparison (heat treatment and non-heat treatment samples), including a broad range of chemical classes. Each annotated metabolite is listed in the Supplementary File S1, together with its abundance and composite mass spectrum.

The first approach was to investigate, using both unsupervised and supervised multivariate statistical analyses, differences between both inoculated samples of *P. fluorescens* 39 taken at two different times, and between inoculated and respective non-inoculated samples. The same comparison was also performed on samples that underwent a final heat treatment. In particular, the underlying goal was to investigate whether the growth of *P. fluorescens* 39 left a distinctive fingerprint in the milk and, if so, whether the heat treatment amplified or masked it.

**Figure 1:**
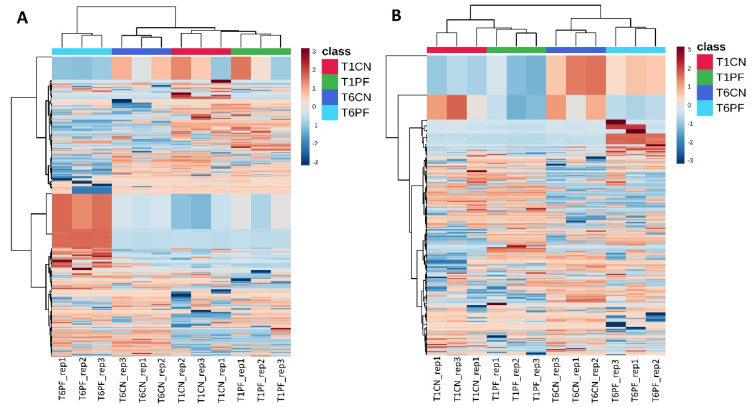
Heatmap-based unsupervised hierarchical cluster analysis (HCA—similarity index: Euclidean; dendrogram linkage method: Ward) of all compounds in the dataset of the non-heat-treated (**A**) and heat-treated (**B**) milk samples. Abbreviations: T1CN = non-inoculated sample incubated for 24 h; T1PF = inoculated sample incubated for 24 h; T6CN = non-inoculated sample incubated for 144 h; T6PF = inoculated sample incubated for 144 h.

Unsupervised clustering was formerly used naïvely to describe relatedness/unrelatedness across treatments, using all annotated compounds. For the non-heat-treated group, the HCA produced by the fold-change-based heatmap led to a substantial separation into two main groups (Figure 1A). One of these consisted of three subgroups—the non-inoculated samples at 24 h and 144 h and the inoculated sample at 24 h—while in a separate group, we found the inoculated sample taken at 144 h. On the other hand, the inoculated samples taken at 144 h were found to possess an exclusive profile compared to the control and other samples, thus revealing an extensive contribution from the *P. fluorescens* 39 metabolic activity. Notably, the unsupervised statistics allowed the two levels of contaminations to be distinguished (Figure 1A), thus confirming that the changes in metabolomic profiles were related to the magnitude of contamination. Regarding the heat-treated samples, a complete separation grouping was observed (Figure 1B). In fact, a separation into two major clusters was observed, with a hierarchically prevalent effect of time of incubation at 6 °C and with an evident discrimination potential of the two combined factors (i.e., contamination level and time incubation at 6 °C). This latter point could be of particular interest to the dairy industries, as it could allow the prior history of incoming milk to be understood.

**Figure 2:**
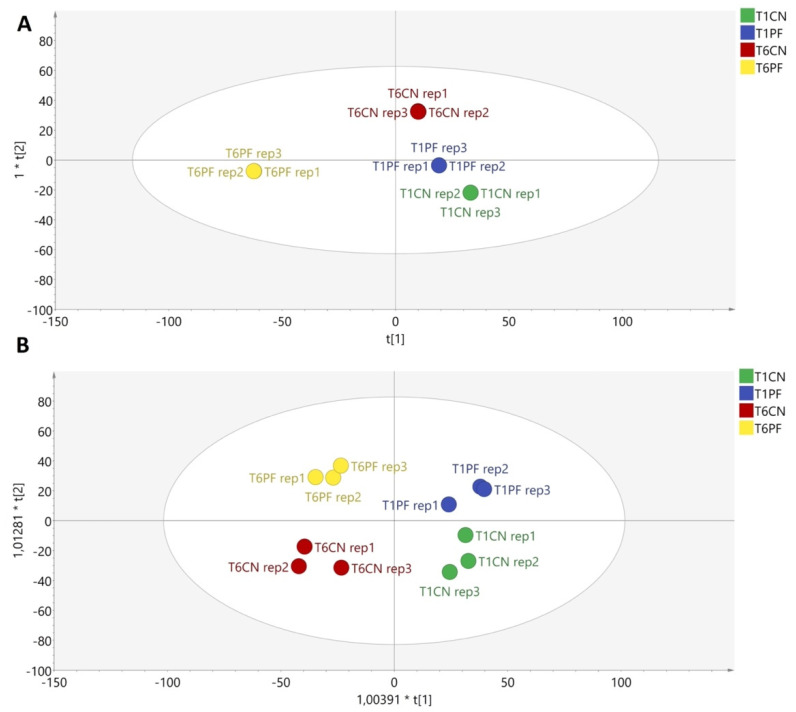
OPLS-DA score plots were generated by considering the metabolomic profile of the non-heat-treated (**A**) and heat-treated (**B**) milk samples. Abbreviations: T1CN = non-inoculated sample incubated for 24 h; T1PF = inoculated sample incubated for 24 h; T6CN = non-inoculated sample incubated for 144 h; T6PF = inoculated sample incubated for 144 h.

After that, multivariate analysis (OPLS-DA) was performed to investigate in a supervised manner the effect of the factors considered (i.e., inoculation, incubation time and heat treatment) on the milk’s metabolomic profile. OPLS-DA is one of the most powerful supervised approaches, adopting an orthogonal signal correction to remove the variation not directly correlated with Y in the X matrix, thus considering only the Y-predictive variation. The resulting OPLS-DA score plots are reported in Figure 2A,B. As can be observed from Figure 2, the OPLS-DA model confirmed the trend previously observed in the HCA heatmaps (Figure 1A,B). Interestingly, regarding the non-heat-treated samples, the different treatments were separated by two latent vectors in the OPLS-DA score plot (Figure 2A). The separation was even clearer in the heat-treated milk samples: the first orthogonal latent vector distinguished the milk samples by storage time (24 vs. 144 h). In contrast, the second vector distinguished inoculated vs. non-inoculated milk (Figure 2B). Therefore, our findings confirm the suitability of untargeted metabolomics to detect potential alterations in milk quality following *Pseudomonas* contamination. Indeed, the goodness parameters of both OPLS-DA models (as reported in Table 2) showed Q^2^ prediction abilities higher than 0.5, as well as good correlations (i.e., R^2^X and R^2^Y). In addition, both models were cross-validated (*p* < 0.001), and overfitting was excluded, with no outliers detected (Supplementary File S2).

### 3.3. Evaluation of the Classes of Marker Compounds Related to P. fluorescens 39 Contamination

**Figure 3:**
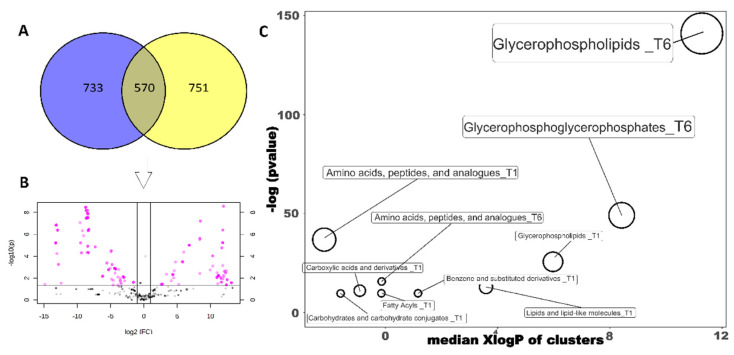
Non-heat-treated sample group; (**A**) Venn analysis built by intersecting compounds of inoculated samples incubated at 24 (blue) and 144 (yellow), using up-regulated metabolites (FC >2) compared to non-inoculated samples incubated 24 and 144 h. (**B**) Volcano plot analysis (*p*-value < 0.05 and FC > 2) on 570 common VIP marker compounds identified at both 24 h and 144 h time points. (**C**) Chemical enrichment analysis generated from the significant compounds: T1 = significant chemical class for inoculated samples at 24 h; T6 = significant chemical class for inoculated samples at 144 h.

Additional data processing was carried out both on non-heat-treated and heat-treated samples to search for classes of marker compounds that could be attributed to the two levels of contamination achieved by *P. fluorescens 39*. Up-accumulated compounds in the inoculated samples versus non-inoculated samples with a fold-change >2 (T1PF vs. T1CN; T6PF vs. T6CN) were considered. Overall, in the case of non-heat-treated samples, 2624 metabolites were found to possess a fold-change value higher than 2 compared to the respective non-inoculated samples. Venn analysis (Figure 3A), however, identified marker compounds common across the two contamination levels. As provided, only 570 out of the 2624 up-accumulated compounds (26.5%) were found in common and were then regarded as being derived exclusively from the metabolic activity of *P. fluorescens 39* in milk. In addition, to identify the most significant compounds of each contamination level, a volcano plot analysis was performed (Figure 3B). Finally, the significant compounds obtained were subjected to enrichment analysis through the online tool ChemRICH class and grouped by chemical classes according to the information reported in the PAMDB.

Overall, as seen in Figure 3C, glycerophospholipids and glycerophosphoglycerophosphates are the most commonly represented compounds of the two levels of contamination, with a substantial increase in samples that underwent a longer incubation time and therefore a higher level of contamination. The phospholipids mentioned above are characteristic of the *Pseudomonas* membrane [29] and, more generally, of most Gram-negative bacteria. The significant membrane phospholipids we found in milk at the lowest contamination level were phosphatidylglycerophosphates, phosphatidylethanolamine and CDP-diacylglycerols, with different combinations of fatty acids in positions C-1 and C-2. In *Pseudomonas*, they are found in both the cytoplasmic membrane and the outer membrane. At the higher level of contamination, in addition to the phospholipids mentioned above, phosphatidylserines, cardiolipins and phosphatidylglycerols were identified. As for the phosphatidylglycerophosphates, these are further characteristic of the *Pseudomonas* membrane.

The same statistical process was applied to heat-treated samples. Glycerophospholipids were also identified as the characteristic class of compounds regardless of the contamination level. In contrast to the non-heat-treated samples, 1682 up-accumulated compounds were found, with 124 common between the two incubation times and the related *P. fluorescens 39* contamination. The subsequent volcano analysis showed considerably fewer significant compounds compared to the non-heat-treated samples. The ChemRICH enrichment analysis confirmed the involvement of glycerophospholipids (particularly at the higher contamination level) (Supplementary File S3; Figure S1).

The most important marker compounds that discriminate between the two levels of contamination are shown in Supplementary File S3. Notably, the phospholipids profile found at the two different contamination levels is not directly attributable to a specific *Pseudomonas* contamination since membrane lipids are quite common to all Gram-negative bacteria, including bacteria of the Enterobacteriaceae family. In this regard, although *Pseudomonas* is rather characteristic of cold-stored milk, it would be interesting to also study other Gram-negative bacteria to see whether the shift in metabolomic signatures might discriminate between different genera or species.

It is also interesting to note that the significant markers of *Pseudomonas* metabolic activity also included protein degradation products, such as dipeptide and tripeptide amino acids, and lipid degradation compounds, such as fatty acids and lipid-derivative compounds. All these markers can be linked to the degradative metabolic activity of *P. fluorescens*. In our case, however, unlike membrane phospholipids, these degradation products did not correlate linearly with the level of contamination.

Overall, our findings are difficult to compare with the existing literature. In fact, to the best of our knowledge, most available work on the subject is based on evaluating the extracellular protease AprX from *Pseudomonas* and its involvement in milk spoilage [30]. Nonetheless, a similar analytical approach using untargeted metabolic profiling was proposed to monitor *Listeria* contamination in milk [31]. In terms of *Pseudomonas* contamination, as reported by [32], a posteriori assessment of contamination in short-time-pasteurized fluid milk continues to be an issue for processors and dairy industries. Considering that broad literature is available regarding the proteolytic and lipolytic activities of *P. fluorescens* in milk, untargeted metabolomics allowed specific metabolite classes related to *P. fluorescens* metabolic activity to be identified, thus indirectly indicating its presence rather than its previous occurrence. This information may prove to be valuable information, complementary to microbiological methods, in investigating the quality of cold-stored milk. Such information can help to properly manage milk quality in order to reduce spoilage processes. Early detection of food spoilage microorganisms is a rather promising tool for food quality monitoring, as reported by Farhana R. Pinu [33]

### 3.4. Confirmation of Proteolysis Markers through Peptidomics

Metabolomics is not a primary choice for detecting markers of *Pseudomonas* proteolysis activity because only the end products (low-molecular-weight compounds, such as amino acids, dipeptides and tripeptides) can be detected. To corroborate the potential of *Pseudomonas*-related proteolytic activity as an indicator of milk quality, a peptidomic approach was also applied. From the peptide abundance matrix, available in the Supplementary File S4, we did not perceive a detectable difference between the peptide profiles of non-inoculated vs. *P. fluorescens* 39-inoculated samples at 24 h of incubation at 6 °C. The peptides found in the inoculated samples—(K)TKVIPYVRYL(-) and (K)VIPYVRYL(-)—are the same as were found in the control sample; this difference is not sufficient to consider them good markers in earlier stages of microbial growth. On the other hand, after an incubation period of 144 h and a contamination level of about 10^8^ CFU/mL, we found a considerable increase in new peptides. Despite confirmation that the result of *Pseudomonas* proteolytic activity offers valuable information in terms of milk quality during cold storage, the sensitivity of untargeted screening is still too poor in this sense. Nevertheless, potential candidate marker peptides for proteolysis and *Pseudomonas* presence could in the future be tested in low-contamination samples using targeted peptidomic analysis to verify whether they are present at lower concentrations.

## 4. Conclusions

The spoilage of milk during cold chain storage is not necessarily directly related to a concurrent growth of microorganisms, and complementary information, including antecedent contaminations, might be useful. In this study, we have shown that the methodology described allowed for detecting *P. fluorescens* metabolomic activity in milk in a simplified lab-scale simulated cold chain using a temperature regime of 6 °C. The untargeted nature of the approach used allowed us to discriminate between non-inoculated and inoculated samples tested after 24 and 144 h of incubation. Notably, the samples analysed after heat treatment provided even clearer metabolomic signatures in which the degree of contamination achieved at different time points was detectable. These outcomes, which allow the identification of previous contamination of mass milk (which is often heat-treated before being processed by the dairy industry), may have potential implications at the industrial level. We identified several classes of marker compounds able to differentiate *P. fluorescens* inoculated milk, with glycerophospholipids and glycerophosphate phospholipids likely providing the most promising information. Future research should be conducted by assessing the validity of our markers under real industrial conditions by increasing sample variability and comparing them with laboratory scale samples to build a dataset of metabolic signatures that could be related to ongoing or previous contamination.

Furthermore, in search of target biomarkers, further research is still needed to better characterise their sensitivity and specificity for *Pseudomonas* rather than other Gram-negative bacteria.

On the other hand, as provided using peptidomics, the peptide profile allowed us to confirm the proteolytic metabolic activity of *Pseudomonas*, in agreement with metabolomics, only when microbial growth is abundant. Therefore, peptide profiles do not yet seem alternative or complementary to metabolomics if early microbial spoilage is to be detected.

## Figures and Tables

**Table 1:** Monitoring of the number of total bacteria count (TBC) and *Pseudomonas* at the point of inoculation, after 24 h and 144 h in milk at 6 °C.

	Non Inoculated Samples			Inoculated Samples		
Culture Monitoring	Time 0 log_10_(CFU/mL)	After 24 h log_10_(CFU/mL)	After 144 h log_10_(CFU/mL)	Time 0 log_10_(CFU/mL)	After 24 h log_10_(CFU/mL)	After 144 h log_10_(CFU/mL)
PAB	<10	<10	<10	4.18 ± 0.06	5.30 ± 0.04	8.64 ± 0.04
TBC	<10	<10	<10	4.26 ± 0.07	5.54 ± 0.15	8.72 ± 0.01

**Table 2:** Goodness-of-fit parameters of the OPLS-DA models built.

Model Parameter	Non-Heat-Treated Milk	Heat-Treated Milk
R^2^X (cum)	0.90	0.58
R^2^Y (cum)	0.99	0.94
Q^2^ (cum)	0.86	0.74

The goodness of fit cumulate (R^2^X(cum) and R^2^Y(cum)) and goodness of prediction cumulate (Q^2^(cum)) values are provided.

## Data Availability

Not applicable.

## Supplementary Materials

The following are available online at https://www.mdpi.com/article/10.3390/foods10061173/s1, Supplementary material S1: Complete list of compounds annotated in cold chain milk samples, either contaminated with *P. fluorescens* or not, using UHPLC/QTOF untargeted metabolomics. Compounds are provided with individual intensities and with composite mass spectra (mass abundance combinations). Supplementary material S2: Validation of the OPLS-DA supervised modelling of the metabolomic profile of cold chain milk samples, either contaminated with *P. fluorescens* or not, at 24 h and 144 h of storage. Hotelling’s T2 analysis for outliers, permutation testing for overfitting and CV-ANOVA cross-validation are provided. Supplementary material S3: List of Variables of Importance in Projection (VIP) marker compounds discriminating between the levels of *P. fluorescens* contamination in the OPLS-DA supervised modelling. Supplementary material S4: Complete list of peptides annotated in cold chain milk samples, either contaminated with *P. fluorescens* or not, using nanoLC/QTOF untargeted peptidomics. Peptides are provided with individual intensities and are grouped by the corresponding protein of origin.
