# Supplementary material for: A Milk Foodomics Investigation into the Effect of Pseudomonas fluorescens Growth under Cold Chain Conditions
Source: Foods. 2021 May 24;10(6):1173. doi: 10.3390/foods10061173 (PMC8225104; doi:10.3390/foods10061173)
Supplement: Supplementary file 1 [file foods-10-01173-s001.zip › Bellassi et al S2 Hotelling's T2, permutation, and CV ANOVA.pdf]

Supplementary file S3: Validation of OPLS-DA discriminant model. Hotelling's T2 using 95% and 99% confidence limits is given in upper panel (A1-non heat treated sample and A2-heat treated sample), whereas outcome of permutation test (N = 200) is given in the lower one (B-non heat treated sample and B2-heat treated sample).

A1

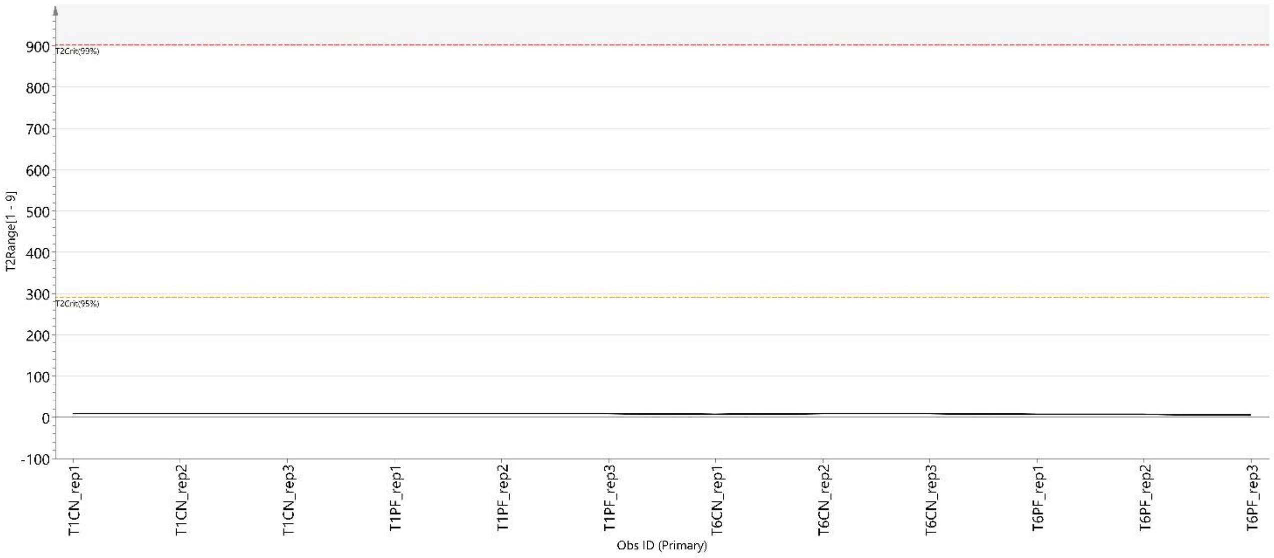

A2

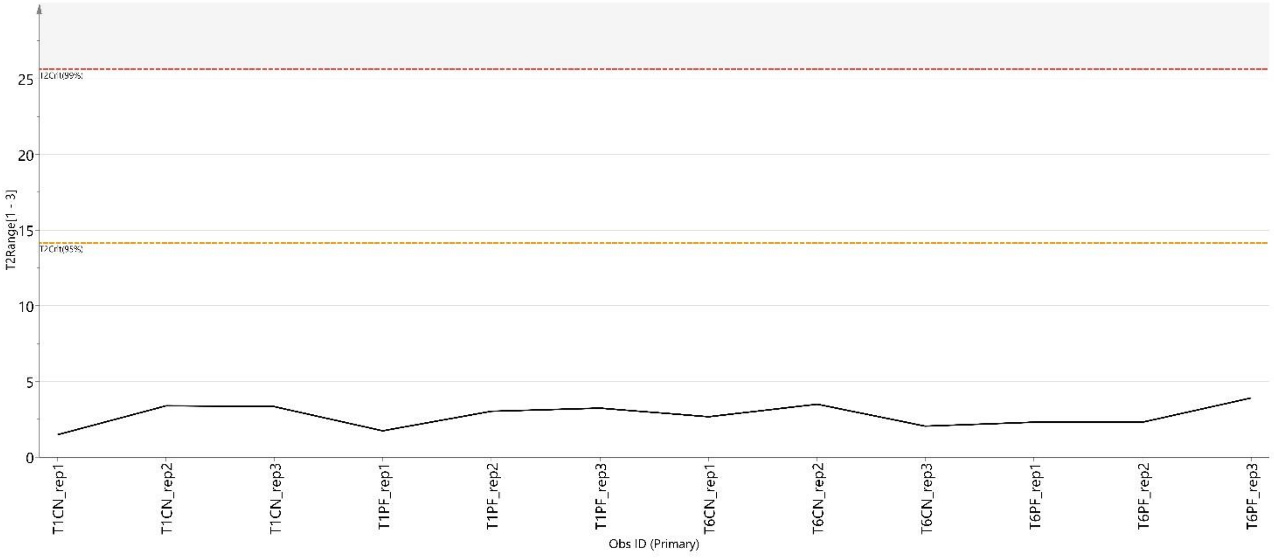

B1

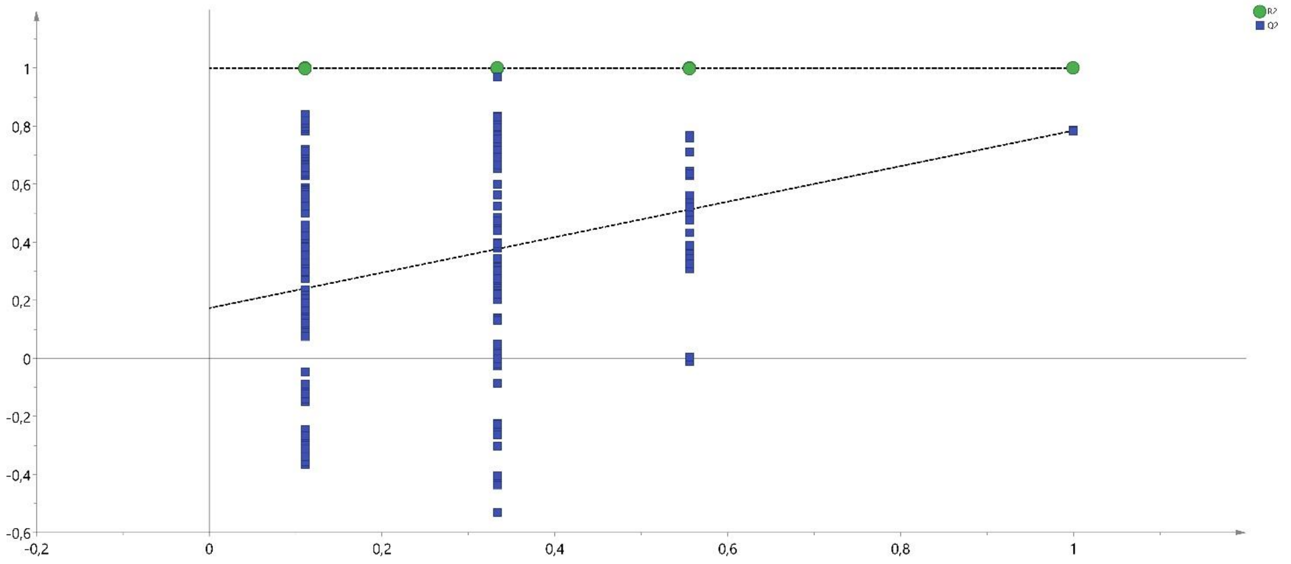
R2
Q2
1
0,8
0,6
0,4
0,2
0
-0,2
-0,4
-0,6
-0,2
0
0,2
0,4
0,6
0,8
1

B2

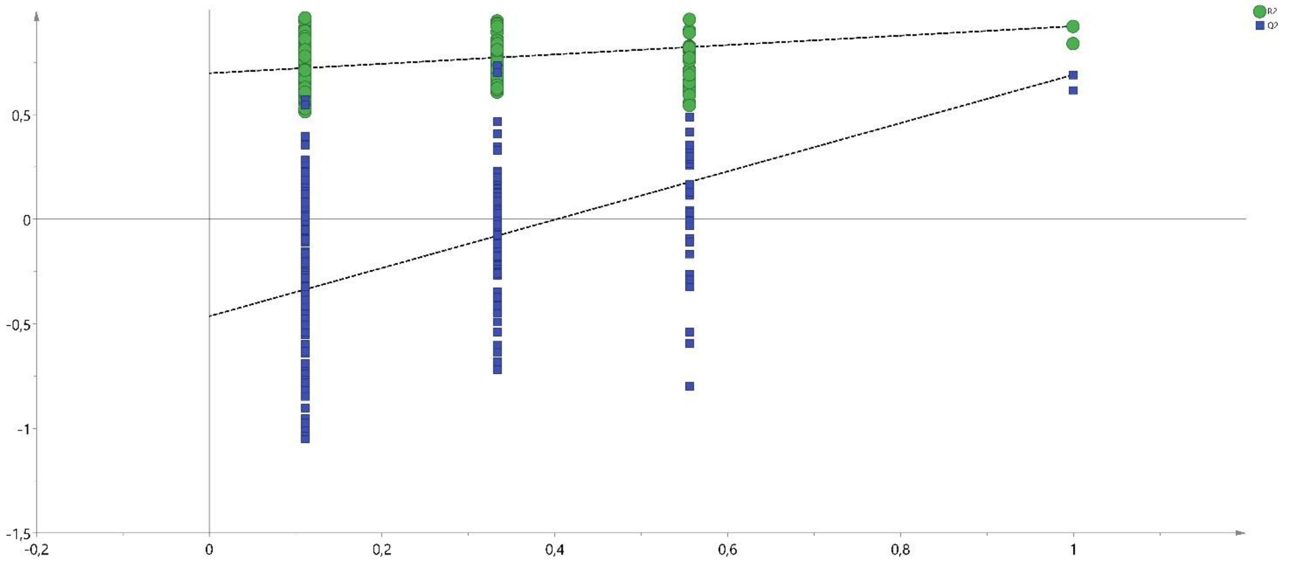
R2
Q2
0,5
0
-0,5
-1
-1,5
-0,2
0
0,2
0,4
0,6
0,8
1
