# Supplementary material for: A Milk Foodomics Investigation into the Effect of Pseudomonas fluorescens Growth under Cold Chain Conditions
Source: Foods. 2021 May 24;10(6):1173. doi: 10.3390/foods10061173 (PMC8225104; doi:10.3390/foods10061173)
Supplement: Supplementary file 1 [file foods-10-01173-s001.zip › Bellassi et al S3 Potential biomarker compounds.pdf]

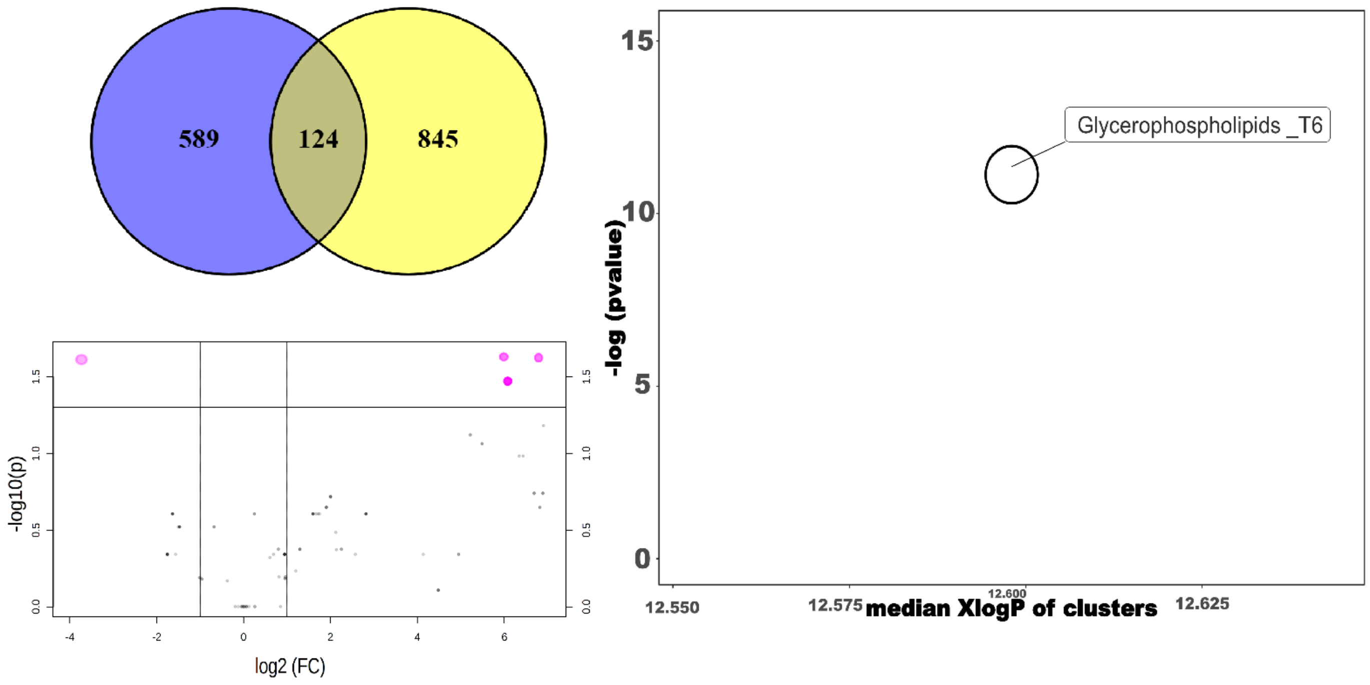

Supplementary 3 FigureS1 Heat treated sample group; (**A**) Venn analysis built by intersecting compounds of inoculated samples incubated at 24 (blue) and 144 (yellow), using up accumulated metabolites (FC >2) compared to non-inoculated samples incubated 24 and 144 h. (**B**) Volcano Plot analysis (p-value < 0.05 and FC > 2) on 570 common VIP marker compounds identified at both 24 h and 144 h time points. (**C**) Chemical Enrichment analysis generated from the significant compounds: T1 = significant chemical class for inoculated samples at 24 h; T6= significant chemical class for inoculated samples at 144 h

Supplementary 3 Table 1 Biomarker compounds from the Volcano analysis (p-value < 0.05; FC value > 2) conducted using the abundance of the compounds relative to the two incubation times, results from UP-accumulated compared to the control (non-inoculated samples), common between the two incubation times (corresponding to the two levels of *P. fluorescens 39* contamination); (**A**) Biomarker compounds Non-heat treated sample group - *P. fluorescens 39* growth after 24 h 6 ℃, (**B**) Biomarker compounds Non-heat treated sample group - *P. fluorescens 39* growth after 144 h 6 ℃, (**C**) Biomarker compounds Heat treated sample group - *P. fluorescens 39* growth after 24 h 6 ℃, (**D**) Biomarker compounds Non-heat treated sample group - *P. fluorescens 39* growth after 144 h 6 ℃.

Score description:

p-value Volcano: Volcano analysis applied to UP accumulated compounds common between T1 and T6 produced as output a pValue expressing a statistical difference between the abundance of compounds in the T1 vs T6 group

VIP score (T6vsT1): Variable importance in projection (VIP) was extracted by conducting an OPLS-DA analysis between UP-accumulated against control(CN) T1 and UP-accumulated against control(CN) T6 compounds.

VIP Standard error: VIP standard error is the standard error of the VIP Score model.

LOG2(FC)(T1PFvsT1CN): This value refers to the FC value for the final compounds and the uninoculated control sample (CN).

A

Non-heat treated sample group -----Pseudomonas **growth after 24 h 6 ℃**						
Database Code	*Class*	*Compound Name*	*p-value Volcano*	*VIP score (T6vsT1)*	*VIP Standard error*	*LOG2(FC)(T1PFvsT1 CN)*
PAMDB0005 82	Alcohols and polyols	3-Dehydroquinate	4.28E-05	1.27246	0.140161	20.33422
BMDB62240	Amino acids, peptides, and analogues	Histidinyl-Proline	6.18E-08	1.27761	0.0594045	12.27888
BMDB62257	Amino acids, peptides, and analogues	Prolyl-Histidine	6.18E-08	1.27761	0.0594045	12.27888
BMDB63626	Amino acids, peptides, and analogues	Glycyl-Isoleucine	9.88E-06	1.27551	0.0925752	15.70902
BMDB00759	Amino acids, peptides, and analogues	Glycyl-L-leucine	9.88E-06	1.27551	0.0925752	15.70902
BMDB00206	Amino acids, peptides, and analogues	N6-Acetyl-L-lysine	9.88E-06	1.27551	0.0925752	15.70902
BMDB00446	Amino acids, peptides, and analogues	N-Alpha-ccetyllysine	9.88E-06	1.27551	0.0925752	15.70902
BMDB00670	Amino acids, peptides, and analogues	Homo-L-arginine	0.000804	1.25087	0.289548	0.33309
BMDB01890	Amino acids, peptides, and analogues	Acetylcysteine	0.003296	1.23151	0.419201	1.9797
BMDB62246	Amino acids, peptides, and analogues	Leucyl-Serine	0.003293	1.2306	0.418402	7.345736

BMDB62262	Amino acids, peptides, and analogues	Threoninyl-Valine	0.003293	1.2306	0.418402	7.345736
PAMDB000189	Amino acids, peptides, and analogues	Ureidosuccinic acid	0.005891	1.22319	0.468699	0.043821
BMDB00821	Amino acids, peptides, and analogues	Phenylacetylglycine	0.006092	1.22243	0.475567	0.010616
BMDB01539	Amino acids, peptides, and analogues	Dimethyl-L-arginine	0.009645	1.20819	0.536462	7.656083
BMDB00267	Amino acids, peptides, and analogues	Pyroglutamic acid	0.014315	1.19781	0.602924	0.559079
BMDB62223	Amino acids, peptides, and analogues	Alanyl-Proline	0.009221	1.19464	0.580857	0.127488
BMDB11175	Amino acids, peptides, and analogues	L-leucyl-L-proline	0.010535	1.18144	0.547645	6.729813
BMDB62574	Amino acids, peptides, and analogues	Pro-Ile	0.010535	1.18144	0.547645	6.729813
BMDB05960	Amino acids, peptides, and analogues	D-Pipecolic acid	0.023623	1.17629	0.649788	6.424854
BMDB00716	Amino acids, peptides, and analogues	L-Pipecolic acid	0.023623	1.17629	0.649788	6.424854
BMDB00070	Amino acids, peptides, and analogues	Pipecolic acid	0.023623	1.17629	0.649788	6.424854
BMDB63628	Amino acids, peptides, and analogues	Phenyl-Leucine	0.039477	1.11473	0.768373	8.06525
PAMDB000305	Benzene and substituted derivatives	4-Nitrophenol	0.00212	1.24628	0.428985	6.846095
PAMDB000914	Benzene and substituted derivatives	3-Carbamoyl-2-phenylpropionaldehyde	0.006092	1.22243	0.475567	0.010616

PAMDB000939	Benzene and substituted derivatives	4-Hydroxy-5-phenyltetrahydro-1,3-oxazin-2-one	0.006092	1.22243	0.475567	0.010616
PAMDB005009	Benzene and substituted derivatives	Benzyl alcohol	0.008693	1.20658	0.539232	1.336482
PAMDB001747	Benzene and substituted derivatives	p-Cresol	0.008693	1.20658	0.539232	7.150248
PAMDB000194	Carbohydrates and carbohydrate conjugates	Nicotinamide riboside	9.24E-05	1.27042	0.145404	21.32872
PAMDB000160	Carbohydrates and carbohydrate conjugates	Gluconic acid	0.004859	1.20642	0.510627	19.2813
PAMDB000057	Carbohydrates and carbohydrate conjugates	Gluconolactone	0.004859	1.20642	0.510627	19.2813
PAMDB001772	Carbohydrates and carbohydrate conjugates	L-Idonate	0.004859	1.20642	0.510627	19.2813
PAMDB000216	Carbohydrates and carbohydrate conjugates	S-Adenosylmethioninamine	0.012316	1.19274	0.600925	0.79528
PAMDB001378	Carboxylic acids and derivatives	L-Ala-gamma-D-Glu-Dap	2.16E-06	1.27678	0.100431	11.72484

PAMDB000267	Carboxylic acids and derivatives	Iminoaspartic acid	0.008628	1.20693	0.537915	0.074889
PAMDB000187	Carboxylic acids and derivatives	Pyrrolidonecarboxylic acid	0.014315	1.19781	0.602924	0.537683
PAMDB000359	Carboxylic acids and derivatives	Pyrroline hydroxycarboxylic acid	0.014315	1.19781	0.602924	0.559079
PAMDB000379	Carboxylic acids and derivatives	Pantetheine 4'-phosphate	0.035834	1.15637	0.739801	9.584125
PAMDB003606	Carboxylic acids and derivatives	pantotheine 4'-phosphate	0.035834	1.15637	0.739801	9.584125
PAMDB100020	Carboxylic acids and derivatives	N-isopropyl-L-glutamine	9.88E-06	1.27551	0.0925752	15.70902
PAMDB003643	Diacylglycerols	DG(12:0/15:0/0:0)	4.1E-07	1.27735	0.0581857	6.651486
PAMDB003659	Diacylglycerols	DG(15:0/12:0/0:0)	4.1E-07	1.27735	0.0581857	6.651486
PAMDB000135	Fatty Acyls	2-Isopropylmalic acid	2.83E-06	1.2767	0.11142	11.4809

PAMDB000641	Fatty Acyls	3-Isopropylmalate	2.83E-06	1.2767	0.11142	11.4809
PAMDB001837	Fatty Acyls	Palmitoleic acid	0.000325	1.26304	0.285528	1.099567
PAMDB000988	Fatty Acyls	D-Galactonate	0.004859	1.20642	0.510627	19.2813
PAMDB000957	Fatty Acyls	7,8-Diaminononanoate	0.008497	1.18366	0.594083	0.66059
PAMDB006552	Glycerophosphoglycerophosphates	PGP(12:0(3-OH)/15:0)	1.75E-05	1.27516	0.0770524	5.595836
PAMDB006586	Glycerophosphoglycerophosphates	PGP(15:0/12:0(3-OH))	1.75E-05	1.27516	0.0770524	5.595836
PAMDB002002	Glycerophospholipids	PE(12:0/19:1(12Z))	2.66E-09	1.27774	0.0714262	7.766574
PAMDB006749	Glycerophospholipids	CDP-DG(10:0(3-OH)/12:0)	5.82E-06	1.27625	0.0861194	7.037282
PAMDB006757	Glycerophospholipids	CDP-DG(10:0/12:0(3-OH))	5.82E-06	1.27625	0.0861194	7.037282

PAMDB006760	Glycerophospholipids	CDP-DG(12:0(3-OH)/10:0)	5.82E-06	1.27625	0.0861194	7.037282
PAMDB006771	Glycerophospholipids	CDP-DG(12:0/10:0(3-OH))	5.82E-06	1.27625	0.0861194	7.037282
PAMDB003935	Glycerophospholipids	PS(10:0(3-OH)/17:0cycw7c)	0.026957	1.15909	0.74802	2.08493
PAMDB003950	Glycerophospholipids	PS(12:0(3-OH)/15:0cyclo)	0.026957	1.15909	0.74802	2.08493
PAMDB003984	Glycerophospholipids	PS(15:0cyclo/12:0(3-OH))	0.026957	1.15909	0.74802	2.08493
PAMDB003995	Glycerophospholipids	PS(17:0cycw7c/10:0(3-OH))	0.026957	1.15909	0.74802	2.08493
PAMDB000032	Glycerophospholipids	Glycerophosphocholine	0.037794	1.1336	0.820864	13.57909
PAMDB006710	Glycerophospholipids	PS(14:0/15:0cyclo)	0.04157	1.13266	0.758625	0.120545
PAMDB006714	Glycerophospholipids	PS(15:0cyclo/14:0)	0.04157	1.13266	0.758625	0.120545

PAMDB006727	Glycerophospholipids	PS(17:0cycw7c/12:0)	0.04157	1.13266	0.758625	0.120545
PAMDB006746	Glycerophospholipids	PS(19:0cycw8c/10:0)	0.04157	1.13266	0.758625	0.120545
BMDB02226	Lipids and lipid-like molecules	Adrenic acid	2.71E-09	1.27773	0.0831744	7.293566
BMDB62590	Lipids and lipid-like molecules	5Z-hexadecenoic acid	0.000325	1.26304	0.285528	1.099567
BMDB12328	Lipids and lipid-like molecules	Palmitelaidic acid	0.000325	1.26304	0.285528	1.099567
BMDB12971	Lipids and lipid-like molecules	1-Hexanol	0.001821	1.23531	0.373137	0.295552
BMDB00847	Lipids and lipid-like molecules	Pelargonic acid	0.036856	1.1113	0.822183	21.24929
BMDB62282	Lipids and lipid-like molecules	Pentyl butanoate	0.036856	1.1113	0.822183	21.24929
PAMDB000850	Naphthalenes	(1R,2S)-Naphthalene 1,2-oxide	0.004458	1.22659	0.447207	17.56378
PAMDB006308	Not Available	N2-succinylglutamate	0.007569	1.2022	0.529412	8.040167
PAMDB003512	Organic phosphoric acids and derivatives	1-Deoxy-L-glycero-tetrulose 4-phosphate	0.017373	1.15201	0.65812	22.97467
PAMDB100092	Organooxygen compounds	2-aminobenzoylacetate	0.004859	1.20642	0.510627	19.2813

PAMDB003527	Prenol lipids	Geranyl diphosphate	0.002413	1.23904	0.37	0.316446
PAMDB000251	Prenol lipids	Octaprenyl diphosphate	0.002413	1.23904	0.37	0.316446
PAMDB000509	Purine nucleotides	DIDP	0.00217	1.23875	0.37844	21.88411
PAMDB000403	Pyrrolopyrimidines	Queuine	0.000714	1.2604	0.254861	0.847293
PAMDB100077	Signaling molecule	N-Butanoyl-D-homoserine lactone	0.005801	1.22356	0.467017	14.65361
PAMDB100073	Signaling molecule	2-nonyl-4-hydroxyquinoline (NHQ)	0.03253	1.15514	0.79634	0.200451
PAMDB005478	Tetralins	tRNA(Tyr)	0.005888	1.22333	0.468036	15.49892
PAMDB000151	Tetrapyrroles and derivatives	Coproporphyrin III	0.00034	1.25966	0.256728	5.76322

B

Non-heat treated sample group -----Pseudomonas **growth after 144 h 6 ℃**						
Database Code	*Class*	*Compound Name*	*p-value Volcano*	*VIP score (T6vsT1)*	*VIP Standard error*	*LOG2(FC)(T1PFvsT1CN)*
BMDB01890	Amino acids, peptides, and analogues_T6	Acetylcysteine	0.032862	1.23151	0.419201	1.9797
BMDB62559	Amino acids, peptides, and analogues_T6	Ile-Ile-Ile-Pro	0.018056	1.19352	0.688296	0.149989
BMDB62232	Amino acids, peptides, and analogues_T6	Glutaminyllysine	0.012877	1.17882	0.607882	6.379116
BMDB62572	Amino acids, peptides, and analogues_T6	Phe-Pro-Ile	0.029662	1.10502	0.724945	7.168326
BMDB62197	Amino acids, peptides, and analogues_T6	N-(2-Oxohexanoyl)homoserine lactone	0.042278	1.06648	0.833997	0.040207
PAMDB001585	Carboxylic acids and derivatives _T6	5,10-Methenyltetrahydrofolate	0.010337	1.19848	0.642769	5.401918
PAMDB000094	Diazines _T6	Orotic acid	0.02777	1.13715	0.626357	4.783832
PAMDB000968	Fatty Acyls _T6	Benzoyl acetyl-CoA	3.38E-07	1.27725	0.091134	0.276173
PAMDB000536	Fatty Acyls _T6	(S)-Hydroxyoctanoyl-CoA	3.84E-07	1.27721	0.092011	0.276173
PAMDB001517	Glycerolipids _T6	1,2-Diacyl-sn-glycerol (ditetradec-7-enoyl, n-C14:1)	0.007938	1.18716	0.557108	0.618484
PAMDB001676	Glycerophosphoglycerophosphates _T6	PGP(14:0/14:0)	6.73E-09	1.27771	0.076302	0.276173
PAMDB006594	Glycerophosphoglycerophosphates _T6	PGP(15:0cyclo/15:0cyclo)	6.41E-09	1.27771	0.07629	0.276173
PAMDB006557	Glycerophosphoglycerophosphates _T6	PGP(12:0(3-OH)/18:1(9Z))	1.2E-08	1.27768	0.076512	0.276173

PAMDB006568	Glycerophosphoglycerophosphates_T6	PGP(14:0(3-OH)/14:0(3-OH))	1.27E-08	1.27768	0.07684	0.276173
PAMDB006573	Glycerophosphoglycerophosphates_T6	PGP(14:0(3-OH)/16:1(9Z))	1.2E-08	1.27768	0.076512	0.276173
PAMDB006609	Glycerophosphoglycerophosphates_T6	PGP(16:1(9Z)/14:0(3-OH))	1.2E-08	1.27768	0.076512	0.276173
PAMDB006625	Glycerophosphoglycerophosphates_T6	PGP(18:1(9Z)/12:0(3-OH))	1.2E-08	1.27768	0.076512	0.276173
PAMDB006539	Glycerophosphoglycerophosphates_T6	PGP(10:0(3-OH)/18:1(9Z))	3.07E-08	1.27761	0.07822	0.276173
PAMDB006555	Glycerophosphoglycerophosphates_T6	PGP(12:0(3-OH)/16:1(9Z))	3.07E-08	1.27761	0.07822	0.276173
PAMDB006608	Glycerophosphoglycerophosphates_T6	PGP(16:1(9Z)/12:0(3-OH))	3.07E-08	1.27761	0.07822	0.276173
PAMDB006624	Glycerophosphoglycerophosphates_T6	PGP(18:1(9Z)/10:0(3-OH))	3.07E-08	1.27761	0.07822	0.276173
PAMDB006545	Glycerophosphoglycerophosphates_T6	PGP(10:0/19:iso)	3.63E-08	1.2776	0.074608	0.276173
PAMDB006650	Glycerophosphoglycerophosphates_T6	PGP(19:iso/10:0)	1.41E-05	1.2776	0.074608	0.276173
PAMDB006409	Glycerophosphoglycerophosphates_T6	PGP(16:1(9Z)/12:0)	4.01E-05	1.27719	0.081166	0.276173
PAMDB006541	Glycerophosphoglycerophosphates_T6	PGP(10:0(3-OH)/19:iso)	1.21E-05	1.27438	0.139491	0.276173
PAMDB006570	Glycerophosphoglycerophosphates_T6	PGP(14:0(3-OH)/15:0)	1.21E-05	1.27438	0.139491	0.276173
PAMDB006649	Glycerophosphoglycerophosphates_T6	PGP(19:iso/10:0(3-OH))	0.007343	1.27438	0.139491	0.276173
PAMDB001677	Glycerophosphoglycerophosphates_T6	PGP(14:1(7Z)/14:1(7Z))	0.003621	1.27083	0.168696	0.276173

PAMDB006538	Glycerophosphoglycerophosphates _T6	PGP(10:0(3-OH)/17:0cycw7c)	0.000047	1.27062	0.163213	0.276173
PAMDB006553	Glycerophosphoglycerophosphates _T6	PGP(12:0(3-OH)/15:0cyclo)	0.000047	1.27062	0.163213	0.276173
PAMDB006591	Glycerophosphoglycerophosphates _T6	PGP(15:0cyclo/12:0(3-OH))	0.000047	1.27062	0.163213	0.276173
PAMDB006613	Glycerophosphoglycerophosphates _T6	PGP(17:0cycw7c/10:0(3-OH))	0.000047	1.27062	0.163213	0.276173
PAMDB006610	Glycerophosphoglycerophosphates _T6	PGP(16:1(9Z)/17:0cycw7c)	0.00852	1.21644	0.573545	0.276173
PAMDB006618	Glycerophosphoglycerophosphates _T6	PGP(17:0cycw7c/16:1(9Z))	0.00852	1.21644	0.573545	0.276173
PAMDB006627	Glycerophosphoglycerophosphates _T6	PGP(18:1(9Z)/15:0cyclo)	0.00852	1.21644	0.573545	0.276173
PAMDB006955	Glycerophospholipids _T6	CL(10:0(3-OH)/12:0/10:0(3-OH)/12:0)	3.38E-09	1.27773	0.075253	0.276173
PAMDB006964	Glycerophospholipids _T6	CL(10:0/12:0(3-OH)/10:0/12:0(3-OH))	3.38E-09	1.27773	0.075253	0.276173
PAMDB006967	Glycerophospholipids _T6	CL(12:0(3-OH)/10:0/12:0(3-OH)/10:0)	3.38E-09	1.27773	0.075253	0.276173
PAMDB006979	Glycerophospholipids _T6	CL(12:0/10:0(3-OH)/12:0/10:0(3-OH))	3.38E-09	1.27773	0.075253	0.276173
PAMDB006954	Glycerophospholipids _T6	CL(10:0(3-OH)/12:0(3-OH)/10:0(3-OH)/12:0(3-OH))	6.33E-09	1.27771	0.075454	0.276173
PAMDB006966	Glycerophospholipids _T6	CL(12:0(3-OH)/10:0(3-OH)/12:0(3-OH)/10:0(3-OH))	6.33E-09	1.27771	0.075454	0.276173
PAMDB003954	Glycerophospholipids _T6	PS(12:0(3-OH)/18:1(9Z))	3.1E-08	1.27761	0.074397	0.276173
PAMDB003970	Glycerophospholipids _T6	PS(14:0(3-OH)/16:1(9Z))	3.1E-08	1.27761	0.074397	0.276173

PAMDB003993	Glycerophospholipids _T6	PS(16:1(9Z)/14:0(3-OH))	3.1E-08	1.27761	0.074397	0.276173
PAMDB004000	Glycerophospholipids _T6	PS(18:1(9Z)/12:0(3-OH))	3.1E-08	1.27761	0.074397	0.276173
PAMDB002026	Glycerophospholipids _T6	PE(19:0cycw8c/12:0)	8.48E-08	1.27747	0.079929	0.276173
PAMDB006675	Glycerophospholipids _T6	PE(16:0/19:0cycv8c)	1.43E-07	1.27742	0.080868	0.276173
PAMDB003898	Glycerophospholipids _T6	PE(16:1(9Z)/19:iso)	1.43E-07	1.27742	0.080868	0.276173
PAMDB002020	Glycerophospholipids _T6	PE(18:0/17:0cycw7c)	1.43E-07	1.27742	0.080868	0.276173
PAMDB001474	Glycerophospholipids _T6	PE(18:1(11Z)/17:0)	1.43E-07	1.27742	0.080868	0.276173
PAMDB006695	Glycerophospholipids _T6	PE(19:0cycv8c/16:0)	1.43E-07	1.27742	0.080868	0.276173
PAMDB003920	Glycerophospholipids _T6	PE(19:iso/16:1(9Z))	1.43E-07	1.27742	0.080868	0.276173
PAMDB006853	Glycerophospholipids _T6	CDP-DG(19:iso/19:0cycv8c)	4.66E-07	1.27711	0.083882	0.276173
PAMDB006953	Glycerophospholipids _T6	CL(10:0(3-OH)/10:0/10:0(3-OH)/10:0)	4.66E-07	1.27711	0.083882	0.276173
PAMDB006963	Glycerophospholipids _T6	CL(10:0/10:0(3-OH)/10:0/10:0(3-OH))	4.66E-07	1.27711	0.083882	0.276173
PAMDB001500	Glycerophospholipids _T6	PG(16:0/19:0)	4.16E-07	1.27709	0.084081	0.276173
PAMDB004086	Glycerophospholipids _T6	PG(16:0/19:iso)	4.16E-07	1.27709	0.084081	0.276173
PAMDB001448	Glycerophospholipids _T6	PG(19:0/16:0)	4.16E-07	1.27709	0.084081	0.276173

PAMDB00 4111	Glycerophospholipids _T6	PG(19:iso/16:0)	4.16E-07	1.27709	0.084081	0.276173
PAMDB00 0798	Glycerophospholipids _T6	PG(18:1(11Z)/16:1(9Z))	4.52E-07	1.27706	0.084458	0.276173
PAMDB00 6501	Glycerophospholipids _T6	PG(16:0/19:1(9Z))	6.04E-06	1.27516	0.113525	0.276173
PAMDB00 1491	Glycerophospholipids _T6	PG(16:1(9Z)/19:0)	6.04E-06	1.27516	0.113525	0.276173
PAMDB00 4090	Glycerophospholipids _T6	PG(16:1(9Z)/19:iso)	6.04E-06	1.27516	0.113525	0.276173
PAMDB00 1485	Glycerophospholipids _T6	PG(17:0/18:1(11Z))	6.04E-06	1.27516	0.113525	0.276173
PAMDB00 1483	Glycerophospholipids _T6	PG(18:1(11Z)/17:0)	6.04E-06	1.27516	0.113525	0.276173
PAMDB00 1488	Glycerophospholipids _T6	PG(19:0/16:1(9Z))	6.04E-06	1.27516	0.113525	0.276173
PAMDB00 6517	Glycerophospholipids _T6	PG(19:0cycv8c/16:0)	6.04E-06	1.27516	0.113525	0.276173
PAMDB00 6525	Glycerophospholipids _T6	PG(19:1(9Z)/16:0)	6.04E-06	1.27516	0.113525	0.276173
PAMDB00 4112	Glycerophospholipids _T6	PG(19:iso/16:1(9Z))	6.04E-06	1.27516	0.113525	0.276173
PAMDB00 3934	Glycerophospholipids _T6	PS(10:0(3-OH)/16:1(9Z))	3.19E-05	1.27378	0.202015	18.07214
PAMDB00 3991	Glycerophospholipids _T6	PS(16:1(9Z)/10:0(3-OH))	3.19E-05	1.27378	0.202015	18.07214
PAMDB00 4102	Glycerophospholipids _T6	PG(19:0cycv8c/19:iso)	0.00005	1.27083	0.180677	0.276173
PAMDB00 4115	Glycerophospholipids _T6	PG(19:iso/19:0cycv8c)	0.00005	1.27083	0.180677	0.276173

PAMDB003948	Glycerophospholipids _T6	PS(12:0(3-OH)/14:0(3-OH))	0.000573	1.2599	0.334872	0.276173
PAMDB003964	Glycerophospholipids _T6	PS(14:0(3-OH)/12:0(3-OH))	0.000573	1.2599	0.334872	0.276173
PAMDB001572	Glycerophospholipids _T6	2-octadecanoyl-sn-glycerol 3-phosphate	0.000569	1.25989	0.282614	14.85499
PAMDB006218	Glycerophospholipids _T6	LPA(18:0/0:0)	0.000569	1.25989	0.282614	14.85499
PAMDB003860	Glycerophospholipids _T6	PE(12:0(3-OH)/19:iso)	0.00042	1.25974	0.308052	0.114885
PAMDB003914	Glycerophospholipids _T6	PE(19:iso/12:0(3-OH))	0.00042	1.25974	0.308052	0.114885
PAMDB006365	Glycerophospholipids _T6	sn-glycero-3-phosphocholine	0.001227	1.25535	0.406735	12.19687
PAMDB003745	Glycerophospholipids _T6	CDP-DG(15:0/19:1(9Z))	0.001275	1.25312	0.406637	0.019423
PAMDB000711	Glycerophospholipids _T6	CDP-DG(16:0/18:1(9Z))	0.001275	1.25312	0.406637	0.019423
PAMDB003757	Glycerophospholipids _T6	CDP-DG(16:1(9Z)/18:0)	0.001275	1.25312	0.406637	0.019423
PAMDB003764	Glycerophospholipids _T6	CDP-DG(18:0/16:1(9Z))	0.001275	1.25312	0.406637	0.019423
PAMDB000726	Glycerophospholipids _T6	CDP-DG(18:1(11Z)/16:0)	0.001275	1.25312	0.406637	0.019423
PAMDB000735	Glycerophospholipids _T6	CDP-DG(18:1(9Z)/16:0)	0.001275	1.25312	0.406637	0.019423
PAMDB003776	Glycerophospholipids _T6	CDP-DG(19:1(9Z)/15:0)	0.001275	1.25312	0.406637	0.019423
PAMDB006805	Glycerophospholipids _T6	CDP-DG(16:0/19:0cycw8c)	0.001495	1.25245	0.416628	0.276173

PAMDB003751	Glycerophospholipids _T6	CDP-DG(16:0/19:1(9Z))	0.001495	1.25245	0.416628	0.276173
PAMDB006809	Glycerophospholipids _T6	CDP-DG(16:1(9Z)/19:0)	0.001495	1.25245	0.416628	0.276173
PAMDB006827	Glycerophospholipids _T6	CDP-DG(18:1(11Z)/17:0)	0.001495	1.25245	0.416628	0.276173
PAMDB006834	Glycerophospholipids _T6	CDP-DG(19:0/16:1(9Z))	0.001495	1.25245	0.416628	0.276173
PAMDB006840	Glycerophospholipids _T6	CDP-DG(19:0cycv8c/16:0)	0.001495	1.25245	0.416628	0.276173
PAMDB003777	Glycerophospholipids _T6	CDP-DG(19:1(9Z)/16:0)	0.001495	1.25245	0.416628	0.276173
PAMDB004050	Glycerophospholipids _T6	PG(12:0(3-OH)/18:1(9Z))	0.001725	1.24974	0.417768	7.727007
PAMDB004066	Glycerophospholipids _T6	PG(14:0(3-OH)/16:1(9Z))	0.001725	1.24974	0.417768	7.727007
PAMDB004089	Glycerophospholipids _T6	PG(16:1(9Z)/14:0(3-OH))	0.001725	1.24974	0.417768	7.727007
PAMDB004096	Glycerophospholipids _T6	PG(18:1(9Z)/12:0(3-OH))	0.001725	1.24974	0.417768	7.727007
PAMDB003966	Glycerophospholipids _T6	PS(14:0(3-OH)/14:0(3-OH))	0.009303	1.21365	0.596249	5.351209
PAMDB003951	Glycerophospholipids _T6	PS(12:0(3-OH)/16:0)	0.006808	1.1907	0.519693	1.665435
PAMDB003965	Glycerophospholipids _T6	PS(14:0(3-OH)/14:0)	0.006808	1.1907	0.519693	1.665435
PAMDB003977	Glycerophospholipids _T6	PS(14:0/14:0(3-OH))	0.006808	1.1907	0.519693	1.665435
PAMDB003988	Glycerophospholipids _T6	PS(16:0/12:0(3-OH))	0.006808	1.1907	0.519693	1.665435

PAMDB003870	Glycerophospholipids _T6	PE(14:0(3-OH)/14:0(3-OH))	0.016662	1.15771	0.683334	0.025551
PAMDB006379	Glycerophospholipids _T6	PG(10:0/10:0)	0.038197	1.15654	0.621711	0.276173
PAMDB001983	Glycerophospholipids _T6	PE(10:0/12:0)	0.016891	1.14799	0.656985	0.069496
PAMDB001993	Glycerophospholipids _T6	PE(12:0/10:0)	0.016891	1.14799	0.656985	0.069496
PAMDB004034	Glycerophospholipids _T6	PG(10:0(3-OH)/19:iso)	0.024397	1.13334	0.78275	13.72789
PAMDB004063	Glycerophospholipids _T6	PG(14:0(3-OH)/15:0)	0.024397	1.13334	0.78275	13.72789
PAMDB004077	Glycerophospholipids _T6	PG(15:0/14:0(3-OH))	0.024397	1.13334	0.78275	13.72789
PAMDB004104	Glycerophospholipids _T6	PG(19:iso/10:0(3-OH))	0.024397	1.13334	0.78275	13.72789
PAMDB003585	Imidazole ribonucleosides and ribonucleotides _T6	1-(5-phosphoribosyl)-5-((5-phosphoribosylamino)methylideneamino)imidazole-4-carboxamide	2.34E-08	1.27765	0.07855	0.276173
PAMDB001692	Organic phosphoric acids and derivatives _T6	Tetradecanoyl-phosphate (n-C14:0)	0.045303	1.08899	0.844302	5.852115
PAMDB001054	Prenol lipids _T6	Undecaprenyl phosphate	1.35E-06	1.27659	0.098523	0.276173
PAMDB001971	Prenol lipids _T6	Ubiquinol-4	5.74E-05	1.27002	0.175978	0.276173
PAMDB001300	Pyrimidine nucleotides _T6	UDP-N-Acetylmuramyl-L-Ala	7.01E-09	1.27771	0.076441	0.276173

C

***Heat treated sample group -----Pseudomonas* growth after 24 h 6 ℃**						
Database Code	*Class*	*Compound Name*	*p-value Volcano*	*VIP score (T6vsT1)*	*VIP Standard error*	*LOG2(FC)(T1PFvsT1CN)*
PAMDB006398	Glycerophosphoglycerophosphates_T1	PGP(10:0/10:0)	0.021678	1.25838	0.635046	5.1064

D

***Heat treated sample group -----Pseudomonas* growth after 24 h 6 ℃**						
Database Code	*Class*	*Compound Name*	*p-value Volcano*	*VIP score (T6vsT1)*	*VIP Standard error*	*LOG2(FC)(T6PFvsT6CN)*
PAMDB006650	Glycerophosphoglycerophosphates_T6	PGP(19:iso/10:0)	0.021678	1.30776	0.345948	10.151
PAMDB006545	Glycerophosphoglycerophosphates_T6	PGP(10:0/19:iso)	0.021678	1.30776	0.345948	10.151
BMDB11175	Amino acids, peptides, and analogues_T6	L-leucyl-L-proline	0.021678	1.31501	0.319884	9.7346
BMDB62574	Amino acids, peptides, and analogues_T6	Pro-Ile	0.021678	1.31501	0.319884	9.7346
PAMDB001600	Glycerophospholipids _T6	CDP-12-Dioctadecanoylglycerol	0.03727	1.28676	0.316725	11.046
PAMDB000736	Glycerophospholipids _T6	CDP-DG(18:1(9Z)/18:0)	0.03727	1.28676	0.316725	11.046
PAMDB000727	Glycerophospholipids _T6	CDP-DG(18:1(11Z)/18:0)	0.03727	1.28676	0.316725	11.046
PAMDB006852	Glycerophospholipids _T6	CDP-DG(19:iso/17:0cycw7c)	0.03727	1.28676	0.316725	11.046
PAMDB000720	Glycerophospholipids _T6	CDP-DG(18:0/18:1(9Z))	0.03727	1.28676	0.316725	11.046
PAMDB006824	Glycerophospholipids _T6	CDP-DG(17:0cycw7c/19:iso)	0.03727	1.28676	0.316725	11.046
